# CRISPR nuclease off-target activity and mitigation strategies

**DOI:** 10.3389/fgeed.2022.1050507

**Published:** 2022-11-10

**Authors:** Beeke Wienert, M. Kyle Cromer

**Affiliations:** ^1^ Graphite Bio, Inc., South San Francisco, CA, United States; ^2^ Department of Surgery, University of California, San Francisco, San Francisco, CA, United States; ^3^ Department of Bioengineering and Therapeutic Sciences, University of California, San Francisco, San Francisco, CA, United States; ^4^ Eli and Edythe Broad Center for Regeneration Medicine, University of California, San Francisco, San Francisco, CA, United States

**Keywords:** Gene therapy, off-target activity, *in vivo* delivery, genome editing, CRISPR/Cas9, next-generating sequencing

## Abstract

The discovery of CRISPR has allowed site-specific genomic modification to become a reality and this technology is now being applied in a number of human clinical trials. While this technology has demonstrated impressive efficacy in the clinic to date, there remains the potential for unintended on- and off-target effects of CRISPR nuclease activity. A variety of *in silico*-based prediction tools and empirically derived experimental methods have been developed to identify the most common unintended effect—small insertions and deletions at genomic sites with homology to the guide RNA. However, large-scale aberrations have recently been reported such as translocations, inversions, deletions, and even chromothripsis. These are more difficult to detect using current workflows indicating a major unmet need in the field. In this review we summarize potential sequencing-based solutions that may be able to detect these large-scale effects even at low frequencies of occurrence. In addition, many of the current clinical trials using CRISPR involve *ex vivo* isolation of a patient’s own stem cells, modification, and re-transplantation. However, there is growing interest in direct, *in vivo* delivery of genome editing tools. While this strategy has the potential to address disease in cell types that are not amenable to *ex vivo* manipulation, *in vivo* editing has only one desired outcome—on-target editing in the cell type of interest. CRISPR activity in unintended cell types (both on- and off-target) is therefore a major safety as well as ethical concern in tissues that could enable germline transmission. In this review, we have summarized the strengths and weaknesses of current editing and delivery tools and potential improvements to off-target and off-tissue CRISPR activity detection. We have also outlined potential mitigation strategies that will ensure that the safety of CRISPR keeps pace with efficacy, a necessary requirement if this technology is to realize its full translational potential.

## Introduction

### Gene therapy and off-target genome editing

Gene therapy to correct, add, or modify genes holds great promise for many genetic disorders, including hemoglobinopathies, immunodeficiencies, and lysosomal storage disorders. Historically, gene therapy referred to viral-mediated gene addition, however this has the potential to disrupt essential genes or activate oncogenes due to semi-random genomic integration (Hacein-Bey-Abina et al., 2008). Gene editing tools such as CRISPR-Cas, TALENs, mega nucleases, or zinc finger nucleases have thus emerged as exciting alternatives due to the ability to target them to specific sites in the genome. Among these, the more straightforward and modular design of CRISPR guide RNAs (gRNA), which direct the Cas protein to a complementary site in the genome, has made them the preferred tool for both research and clinical applications. Ongoing clinical trials using CRISPR-modified cells have published results without any adverse events for both genome editing in T cells (Lu et al., 2020; Stadtmauer et al., 2020) and hematopoietic stem and progenitor cells (HSPCs) (Frangoul et al., 2021). In addition, the first clinical trial using CRISPR-Cas9 to treat transthyretin amyloidosis by editing hepatocytes *in vivo* has reported disease phenotype improvements in a small group of patients (Gillmore et al., 2021). These early clinical trials highlight the immense potential of CRISPR-Cas9 to treat disease, albeit lacking long-term follow-up data to support safety in humans.

**FIGURE 1:**
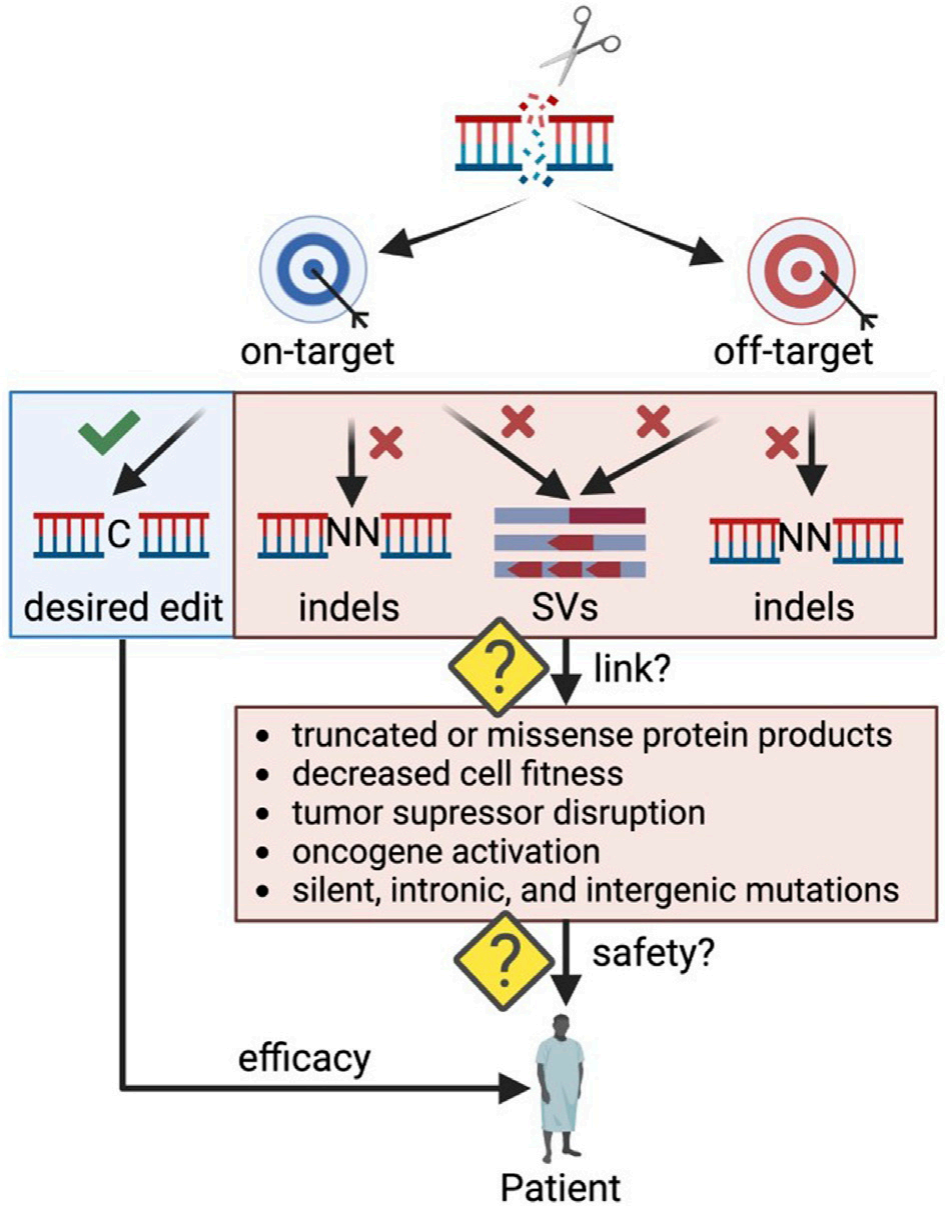
Outstanding questions to ensure safety in therapeutic genome editing applications. Indels: insertions and deletions; SVs: structural variants. Created with BioRender.com.

One concern of clinical genome editing is the potential to cause unintended DNA alterations that may have a detrimental effect on cellular function (Figure 1). These undesired consequences can stem from on-target or off-target edits causing unwanted insertions and deletions (indels) or larger rearrangements (structural variants (SVs)) such as translocations, inversions, and duplications. The field has made great progress in developing methods to detect undesired editing events *in silico*, in cell-free DNA *in vitro*, and in live cells *ex vivo* (Blattner et al., 2020), but often it is challenging to link genomic alterations to their impact on cellular health and function. For example, off-target indels occurring in a gene desert may have no phenotypic effect, while some indels at the on-target site may lead to aberrant mRNA and protein products (Tuladhar et al., 2019) that significantly impact cell function (Lindeboom et al., 2019). As CRISPR-Cas9 genome editing moves towards *in vivo* therapeutic applications, making this link becomes even more critical as rare events could be detrimental if occurring in an oncogenic context. In addition, *in vivo* applications carry the risk of both on- and off-target genome edits in an unintended cell type such as the germline or other tissues.

This field is rapidly evolving and new technologies to quantify unintended modifications are continuously being developed and evaluated. Furthermore, there is no broad consensus on the most appropriate measures needed to be taken to comprehensively assess the frequency and risks of CRISPR off-target activity, both for publication in high-impact journals or pre-clinically for the FDA. This review will therefore summarize the state of the field in terms of the current methods to evaluate on- and off-target gene edits, recent advances in method development for both *ex vivo* and *in vivo* editing workflows, and strategies for mitigation and reduction of off-target and off-tissue edits altogether.

### Methods to find off-targets sites in genome editing applications

**FIGURE 2:**
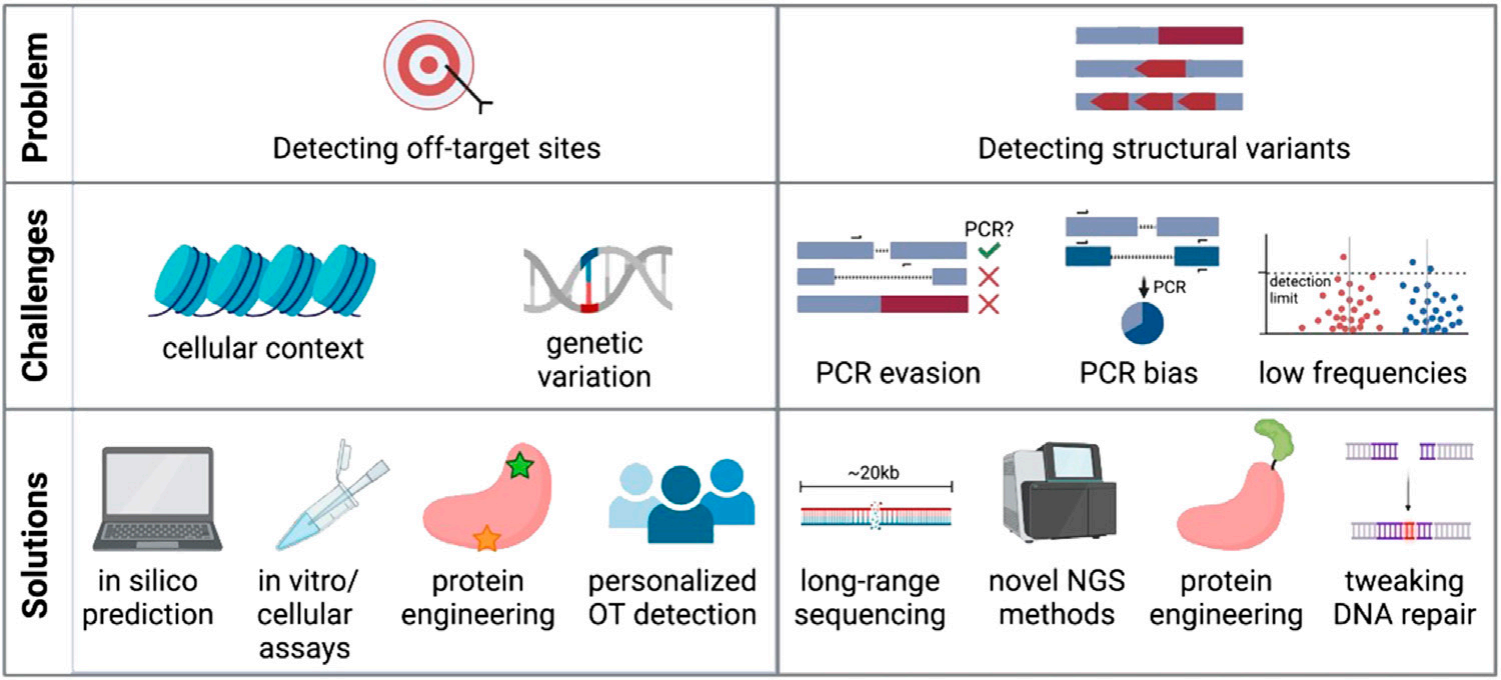
Challenges and potential solutions of current off-target detection methods. Created with BioRender.com.

One main advantage of CRISPR-Cas editing over viral genome addition is that it is specifically targeted to a gene locus rather than dependent on random integration. However, as the genome encompasses billions of base pairs, it is possible that the CRISPR-targeted sequence has a near-match elsewhere. Indeed, it has been shown that Cas9 and other nucleases will often cut highly homologous sequences depending on the location of the mismatch and the genomic context (Mali et al., 2013a; Fu et al., 2013; Hsu et al., 2013; Pattanayak et al., 2013). Finding these off-target sites is critical so that the risk of unintended genomic events can be assessed and minimized (Figure 2).

Many computational tools are available to identify highly homologous genomic sequences and thus predict potential off-target sites for CRISPR-Cas9 activity (Bae et al., 2014; Cradick et al., 2014; Heigwer et al., 2014; Montague et al., 2014; Xiao et al., 2014; Zhu et al., 2014; Singh et al., 2015; Stemmer et al., 2015; Concordet and Haeussler, 2018; Aprilyanto et al., 2021). The application of machine learning to large experimental datasets has further improved the predictive power of these bioinformatic tools (Allen et al., 2018; Shen et al., 2018; Xiang et al., 2021), although these algorithms are biased towards the input gRNAs and reference genomes used to build their predictions. After identifying possible regions of off-target activity, screening must be done in genome-edited cells to confirm whether these sites show signs of CRISPR activity. This is most often done using targeted sequencing of candidate sites with standard sequencing panels achieving detection of variants at or below 5% frequency (Starks et al., 2021), while the detection limit for indels might be lower (0.2-1%) depending on sequencing depth (Chaudhari et al., 2020).

Alternatively, a number of experimental methods have been developed to find off-target sites that may not have been bioinformatically predicted based on homology to the gRNA. These methods vary widely in their approach and even starting material, using cell-free genomic DNA *in vitro* (Kim et al., 2015; Cameron et al., 2017; Tsai et al., 2017; Kim and Kim, 2018; Lazzarotto et al., 2020), in intact live cells *ex vivo* (Crosetto et al., 2013; Tsai et al., 2015; Yan et al., 2017; Wienert et al., 2019; 2020; Zhu et al., 2019; Dobbs et al., 2022), and *in vivo* animal models (Akcakaya et al., 2018; Wienert et al., 2019; Liang et al., 2022). Methods that use cell- and nucleosome-free DNA generally report the highest number of off-targets, many of which cannot be verified in a cellular context (Cromer et al., 2022a). Furthermore, methods such as GUIDE-Seq have been shown to identify more off-target sites in immortalized cell lines than when assaying primary cells (Shapiro et al., 2020). This highlights the importance of chromatin context and DNA repair factors in determining therapeutically relevant off-target activity. And even when using intact cells as input into these assays, conclusions drawn from immortalized cell lines with accumulated variants, distorted karyotypes, and dysfunctional DNA repair pathways (Mittelman and Wilson, 2013; Passerini et al., 2016) may confound the clinical relevance of identified off-target edits.

In spite of these myriad strategies for detection of off-target indels, recent work has shown that *ex vivo* editing in HSPCs elicits very few bona fide off-target sites (<1 true off-target site per gRNA) when using clinically relevant workflows (Cromer et al., 2022b). Of bona fide sites, all were highly homologous to the target sequence and predicted by the majority of *in silico* methods included in the study. However, several sources have shown that genetic variation amongst people can impact off-target activity (Lessard et al., 2017; Lazzarotto et al., 2020; Cromer et al., 2022a). Therefore, implementing a personalized patient-specific workflow in gene therapy products may be needed to circumvent the issue. Common SNPs can be taken into account for *in silico* prediction, *in vitro* methods could be personalized by using genomic DNA extracted from a patient sample (Tsai et al., 2017; Lazzarotto et al., 2020), and some cellular methods are amenable to use on primary cells from patients (Wienert et al., 2019). However, this type of personalized off-target analysis is limited by cost, logistical feasibility, and the availability of patient material.

Taken together, researchers have a broad range of tools available that allow them to identify potential off-target sites *in silico*, in cell-free DNA *in vitro*, in live cells *ex vivo*, and in animal models. By applying these tools sensibly in the experimental design of therapeutic genome editing strategies, off-target gene editing can be identified, and measures can be taken to minimize these unintended events.

### Methods to detect structural variation at double-strand breaks

While the off-target detection methods described above are most useful for identifying localized effects of DNA double-strand breaks (DSBs), larger scale off-target effects have been observed. These include gross chromosomal rearrangements such as translocations (Bothmer et al., 2020; Stadtmauer et al., 2020; Samuelson et al., 2021), chromothripsis (Leibowitz et al., 2021), and aneuploidy (Amendola et al., 2022; Nahmad et al., 2022). Translocation events most often occur as a consequence of: 1) on-target cleavage and recombination with a homologous region of the genome (Turchiano et al., 2021); 2) simultaneous cleavage at an on-target and off-target sequence (Lattanzi et al., 2021); or 3) following multiple on-target cleavage events in multiplexed editing workflows (Qasim et al., 2017; Bothmer et al., 2020; Stadtmauer et al., 2020; Samuelson et al., 2021; Diorio et al., 2022). In addition, large-scale deletions either surrounding the cut site or of the distal end of the chromosome can occur (Cullot et al., 2019), as well as copy-neutral loss-of-heterozygosity (Boutin et al., 2021).

Although targeted amplicon sequencing is commonly used to report on small indels at the cut site, most standard sequencing methods only allow sequencing of relatively short amplicons (hundreds of base pairs). Detecting and quantifying large-scale, multi-kilobase events with PCR-based sequencing methods thus remains challenging (Figure 2B). This is due to several reasons: 1) any deletion that eliminates primer binding sites would not be efficiently amplified with standard sequencing methods and would be missed; 2) if the primer binding sites are preserved larger deletions could skew the PCR reaction towards shorter amplicons and overestimate deletion events; and 3) other undesired on-target events may include inversions, gene duplications, and large insertions (Skryabin et al., 2020) that may also evade detection by PCR-based methods. New No-Amp long-range sequencing protocols avoid PCR and instead use CRISPR-Cas9 to enrich for the sequence of interest up to tens of kilobases. This PCR-free strategy circumvents size bias and can identify large deletions and other structural variants at the target site. However, limited read depth can make it difficult to detect and quantify low-frequency events and low base-calling accuracy of some methods may not achieve single base pair resolution (Lang et al., 2020). However, if these sequencing methods are able to improve and become cheaper, they may become the standard for evaluating structural variants after genome editing.

In addition to large deletions, other genomic abnormalities remain technically challenging to capture, especially when occurring at low frequency. To identify translocations of the on-target site with other genomic regions, several assays have been developed (Zheng et al., 2014; Hu et al., 2016; Qasim et al., 2017; Giannoukos et al., 2018; Yin et al., 2019; Turchiano et al., 2021) which use a sequence at the on-target site as “bait” and next-generation sequencing and bioinformatics to identify the “prey.” This allows identification of genomic sequences that have been fused to the on-target site. Another bioinformatic approach analyzes multiplexed-PCR data for on- and off-target sites using a pipeline specific for translocation detection (Amit et al., 2021) which could allow these events to be quantified from pre-existing data.

The delivery modality of the genome editing tools can also introduce unintended effects. For instance, if the nuclease or DNA repair template is delivered by adeno-associated virus (AAV), there is the possibility that non-homologous integration of inverted terminal repeats (ITRs) could occur (Hanlon et al., 2019; Nelson et al., 2019), however this may have minimal effect on adjacent genes if the template is promoter-less. The introduction of DSBs by a nuclease increases the amount of non-homologous integration of AAV vector sequences (Miller et al., 2003; Miller et al., 2004), however the overall frequency seems to be determined by the genomic context and can range between 0.06% and 12.5% of total events (Hanlon et al., 2019). These rare events can be captured with long-range sequencing methods or by a recently developed next-generation sequencing method, named ITR-Seq (Breton et al., 2020), which can identify and quantify ITR integrations on a genome-wide basis independent of the on-target site.

In summary, the field has made great progress in developing methods that can identify structural variants including deletions, inversions, duplications, insertions, and translocations. However, absolute quantification of these events remains challenging due to their low frequency of occurrence. While promising, novel long-range sequencing strategies are still lacking read depth and quality compared to traditional sequencing methods. As structural variants are diverse it is currently not possible for a single assay to capture all possible events, but future advances in sequencing technology could allow for this to become a reality.

### Methods to identify unintended editing events *in vivo*

Most CRISPR-based therapies currently in the clinic rely on isolation of patient-derived stem cells, *ex vivo* modification, and re-transplantation. This approach thereby addresses the limited availability of matched donors and risk of immune rejection or graft-versus-host-disease associated with allogeneic transplantation. However, these strategies are only compatible with cell types that may be safely isolated, modified *ex vivo*, and transplanted back into the patient, such as HSPCs. Therefore, the next frontier will be to deliver genome-editing components to modify cells directly where they reside in the body.

Toward this end, many delivery modalities have been developed and optimized to transduce clinically relevant cell types *in vivo* (Long et al., 2016; Goldstein et al., 2019; Mangeot et al., 2019; Gillmore et al., 2021). These platforms are now being used to package and deliver CRISPR-based editing tools *in vivo*, which has shown initial success in the first human clinical trials. One of the most prominent of these trials was conducted by Intellia where a liver-tropic lipid nanoparticle (LNP) was used to deliver Cas9 mRNA along with a gRNA specific to the *TTR* gene in order to treat transthyretin amyloidosis (Gillmore et al., 2021). This strategy effectively lowered serum TTR levels up to 87% from baseline in human patients, serving as a landmark study for efficacy of Cas9 to achieve a clinical endpoint. While *in vivo* Cas9 delivery was found to be quite effective in this instance, there was limited data collected to confirm safety aside from the absence of severe adverse events in these patients.

**FIGURE 3:**
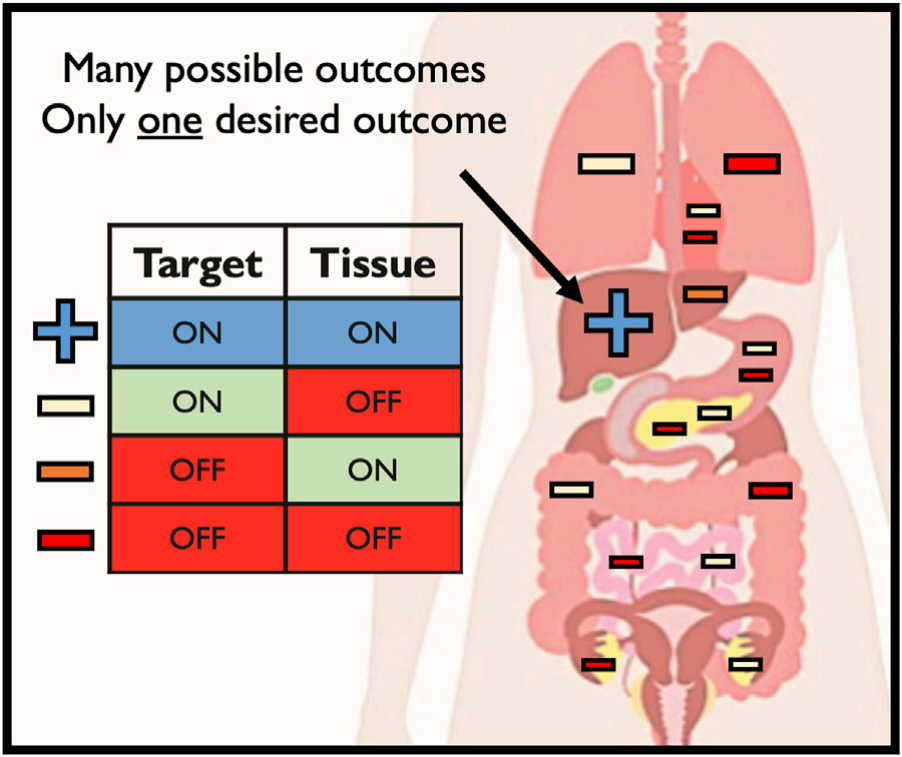
Possible outcomes of in vivo genome editing.

When delivering editing tools *in vivo*, there is only a single desired outcome—on-target editing at the intended CRISPR cleavage site in the intended target tissue (Figure 3). However, a number of unintended consequences can occur following delivery of editing tools to patients *in vivo*, such as: 1) off-target genomic activity in the intended target tissue (off-target, on-tissue); 2) on-target genomic activity in unintended tissue types (on-target, off-tissue); and 3) off-target genomic activity in unintended tissue types (off-target, off-tissue). Off-tissue events in the gonads are of particular clinical and ethical concern since these could result in changes to the germline which may be transmitted to a patient’s offspring (Turocy et al., 2021). Despite these fears and the use of methods to detect off-target Cas9 activity *in vivo* in animal models (Akcakaya et al., 2018; Wienert et al., 2020; Liang et al., 2022), no study to date has investigated the frequency of unintended events following delivery of editing tools to human patients in a clinical context *in vivo.* In the seminal TTR Cas9-LNP paper, the only investigation into off-target activity was done by performing GUIDE-Seq *ex vivo* in hepatocytes (Gillmore et al., 2021). While this is helpful in locating sites of potential off-target activity in the patient’s genome, these results were not validated *in vivo*. In the simplest form, clinically routine liver biopsies could have been performed pre- and post-delivery (perhaps at sites both near and far from the hepatic artery where the LNP would have entered the liver) to quantify the frequency of on- and off-target activity at the on-tissue site. However, this approach would yield little insight into CRISPR activity outside the liver, even though this LNP was reported to edit the spleen, adrenal glands, and ovaries at detectable frequencies. While the liver may be easily biopsied, this is not a routine procedure for many other tissues, particularly the ovaries. This therefore presents a major barrier to ensuring the safety of *in vivo* CRISPR delivery.

A potential source of genetic material that could be obtained in a minimally invasive fashion to determine the frequency of intended and unintended editing events following *in vivo* CRISPR delivery is cell-free genomic DNA (cfDNA). CfDNA is primarily derived from dying cells that release their genomic material into the bloodstream. Because of this, assaying cfDNA may be a powerful approach for detecting potentially pathogenic effects of CRISPR delivery, either in terms of genotoxic or oncogenic editing events. While cfDNA has primarily been used in the diagnostic space to detect occurrence/relapse of cancer (Bronkhorst et al., 2019), this technology is sensitive enough to sample maternal blood to discover *de novo* mutations in the fetus during pregnancy (Kitzman et al., 2012). In fact, a proof-of-concept study used cfDNA to map insertion sites following *in vivo* delivery of lentiviral vectors (Cesana et al., 2021). Therefore, a comparable strategy could be employed to quantify the frequency of on- and off-target cleavage activity following *in vivo* CRISPR delivery. However, unlike the workflow developed for mapping lentiviral insertions which relies on sequencing outward from the vector backbone, mapping sites of CRISPR activity may be aided by defining high-likelihood regions of activity. This could be done using *in silico* or empirical off-target detection methods defined above, and candidate regions could then be probed for indels by targeted deep sequencing of cfDNA. An alternative approach that would not require defining CRISPR off-target sites *a priori* could be to use translocations as a surrogate for on- and off-target activity by adapting technologies such as LAM-HTGTS (Hu et al., 2016), CAST-Seq (Turchiano et al., 2021), or PEM-Seq (Yin et al., 2019) to use patient cells or cfDNA as input. Furthermore, if using cfDNA isolated from peripheral blood is successful, a similar approach could be used to detect occurrence of CRISPR activity in cerebrospinal fluid (CSF) as well to quantify the ability of genome editing tools to cross the blood-brain barrier and edit cells residing in the brain, which may not be safely biopsied.

While cfDNA presents an opportunity to quantify on-target and off-target editing, it may give little insight into the tissue of origin. To shed light on this without invasive biopsies, the use of cell-free RNA (cfRNA) is a possibility. Analogous to the use of cfDNA, workflows to isolate cfRNA from the peripheral blood have been developed that allow insight into the tissue of origin due to the predominant expression patterns of cells releasing DNA and RNA into the bloodstream (Cheung et al., 2019). While the degree to which tissue-of-origin could be gleaned from this approach has yet to be fully explored, the investigation of on- and off-target CRISPR activity at expected cleavage sites in cfRNA could determine whether intended or unintended genome editing results in changes to the transcriptome. Since sites of CRISPR off-target activity typically reside in intergenic regions of the genome with no known functional significance (Cromer et al., 2022b), it is possible that CRISPR activity will be detected in cfDNA, but not in transcribed cfRNA. This could be an important measure to assay efficacy and safety of *in vivo* editing immediately following therapeutic delivery as well as over time. The combined cfDNA/RNA approach could also be an effective way to detect pathogenic clonal expansion of edited cells following treatment. In this specific use case, it may not be necessary to have identified the initiating driver genomic event, but even oligoclonality of passenger events—such as a particular indel at the on- or common off-target sites—could allow us to infer that clonal expansion is occurring. Importantly, the strategies proposed are most likely to capture and monitor the frequency of small site-specific indels, and more sophisticated methods (like those described earlier) may be needed to identify large genomic aberrations in cfDNA/RNA.

### Linking genomic outcomes to off-target significance

Even when we can successfully identify off-target CRISPR effects, determining if an unintended editing event is of concern to the patient’s health remains challenging (Figure 1). Broadly speaking, off-target genomic events are most likely to either elicit no effect or result in a loss or gain of fitness. While loss of fitness will likely result in drop out of the cell carrying the undesired event, gains of fitness are of greater concern due to the possibility of oncogenicity. Although site-specific off-target effects are infrequent (Cromer et al., 2022b), in the event that they do occur, the likelihood of directly disrupting another gene is small (only 1% of the human genome is coding DNA and only 7.2% of predicted off-target sites for exon-targeting gRNAs fall in exonic regions). And while modifications to non-coding DNA sequences may alter gene expression patterns or modify elements with as-yet-unknown important functions (ENCODE Project Consortium, 2012) interpreting non-coding genomic disruptions is difficult. As our understanding of the function of non-coding regions of DNA improves, we may better predict the impact of off-targets modifications in the future. Until then, we must rely on methods that can measure oncogenicity and toxicity from off-target modifications events *in vitro* or *in vivo*.

The most conventional approach to assess tumorigenicity of cell products is implanting cells at an ectopic site in immunodeficient mice followed by monitoring for tumor growth and other adverse events (Human Gene Therapy Products Incorporating Human Genome Editing | FDA). One major caveat of this method is that it has limited sensitivity, depends on the animal model used, and may miss low frequency events (Sato et al., 2019). Alternative *in vivo* approaches have developed technology to track clonality of cell-based products following *ex vivo* HSPC editing and transplantation through barcodes included in the HDR template (Ferrari et al., 2021; Sharma et al., 2021) or by tracking indel diversity (Magis et al., 2022). These technologies can identify clones that have expanded abnormally and hint towards genomic events that led to the oncogenic transformation. Currently these approaches have been limited to research applications but could potentially also be incorporated in therapeutic workflows in the future. However, *in vivo* studies are time- and cost-intensive and can slow down the drug development process immensely. Thus, *in vitro* studies that measure oncogenicity or genomic instability would be preferred, though these may not properly recapitulate *in vivo* activity. While performing whole genome sequencing on every cell product for every patient would ensure an unbiased approach of variant discovery across the whole genome, the currently limited coverage per base pair would miss low-frequency events. Using an intermediate approach of exome sequencing the most commonly mutated oncogenes and tumor suppressors increases read depth significantly and could provide a feasible alternative to assess the safety of *ex vivo* gene therapy drug products (Cromer et al., 2022a).

While the above work focuses on unintended off-target effects, even unintended *on*-target effects can lead to adverse effects. For instance, when a therapeutic editing approach targets a coding sequence—like knocking out a pathogenic gene to correct a disease phenotype (Gillmore et al., 2021)—an array of indels will form at the cut site. A recent report has shown that Cas9-induced indels can result in the formation of disrupted, non-natural mRNAs, which can be translated into aberrant protein products (Tuladhar et al., 2019). This study found that indels can induce internal ribosome entry sites to produce alternative mRNAs or induce exon skipping by disrupting exon-splicing enhancers. The same study also provided a bioinformatic tool to help design gRNAs to avoid such events (Tuladhar et al., 2019). Since truncated protein products could exert a dominant negative function (Savas et al., 2006), potential undesired translated proteins should be studied carefully. Properly characterizing the genome-edited cell population by combining on-target amplicon sequencing with mRNA sequencing and proteomics may allow us to identify and develop strategies to reduce the occurrence of such events.

Taken together, linking genomic events to oncogenicity is difficult and currently available *in vitro* and *in vivo* assays often lack sensitivity. Progress has been made to develop barcoding technologies that can track transformed cells and next-generation sequencing methods such as exome and RNA sequencing can also help identify oncogenic events.

### Approaches to reduce off-target and off-tissue editing

**FIGURE 4:**
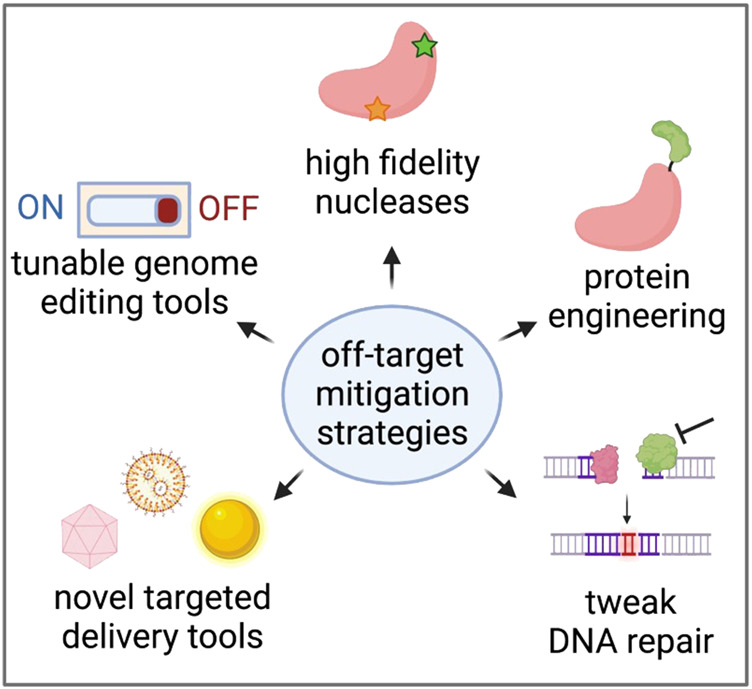
Strategies to reduce unintended genome editing events. Created with BioRender.com.

As we learn more about the types of editing events that can occur at on- and off-target sites, many researchers are developing methods to reduce off-target effects altogether. These efforts range from protein engineering to make nucleases more specific to the discovery of novel, more specific delivery vehicles of genome editing reagents *in vivo* (Figure 4).

Careful nuclease selection and gRNA selection is often the first step when designing a *de novo* genome editing strategy (Lee et al., 2016). While a number of CRISPR nucleases have been discovered (Swarts and Jinek, 2018; Li and Peng, 2019), the majority of clinical efforts to date have used one of the original enzymes characterized, *Streptococcus pyogenes* Cas9 (SpCas9) (Jinek et al., 2012). This nuclease is one of the most common due to its relatively unrestrictive protospacer adjacent motif (PAM) and its high frequency of cleavage activity at a wide variety of loci in a range of cell types across a number of organisms, from humans to Arabidopsis (Mali et al., 2013b; Miki et al., 2018). While this nuclease typically has few genomic sites of off-target activity, some of these can be cut at high frequencies (>30% of alleles harboring indels), depending on the specificity of the particular gRNA (Cromer et al., 2022a). To address this, more specific versions of Cas9 have been engineered which reduce off-target activity by > 20-fold (Chen et al., 2017; Vakulskas et al., 2018; Bravo et al., 2022; Kulcsar et al., 2022). In doing so, incorporation of these higher-fidelity Cas9 variants has been shown to reduce the risk of large-scale genomic rearrangements (Turchiano et al., 2021). In addition to engineering more specific nucleases, a study that fused Cas9 to the exonuclease TREX2 in order to prevent perfect DNA repair reported significantly fewer large deletions and nearly eliminated chromosomal translocations during multiplex editing in T cells (Yin et al., 2022).

The format in which Cas9 is delivered—most often DNA, mRNA, or ribonucleoprotein (RNP)—will alter Cas9 expression and duration of exposure. This in turn has been shown to impact off-target activity, with short half-life RNP and mRNA resulting in lower off-target activity than longer-lived DNA formulations (Cameron et al., 2017; Lu et al., 2019; Zhang et al., 2021). In addition, tunable/inducible control strategies have been incorporated to regulate CRISPR expression using bioavailable small molecules, (Truong et al., 2015; Zhao et al., 2018), light (Nihongaki et al., 2015), and even magnets (Hsu and Hu, 2019). Similarly, other groups have developed self-inactivating Cas9 and AAV delivery vectors that may prevent prolonged exposure to genome editing tools and therefore reduce the likelihood of unintended activity or genomic events (Li et al., 2019; Ibraheim et al., 2021). However, depending on the tools these strategies are applied to, there remains the potential for off-target activity or large-scale genomic rearrangements following creation of DSBs. Furthermore, in their current forms, most approaches are only compatible with *ex vivo* editing workflows where high efficiency delivery of large payloads is possible.

All nuclease-based genome editing applications rely on DSB resolution, therefore modifying the cell’s natural DNA damage repair pathways has emerged as a strategy for increasing the ratio of intended to unintended genomic events (Yeh et al., 2019; Xue and Greene, 2021). For example, transiently inhibiting non-homologous end joining (NHEJ)-mediators such as 53BP1 or DNA-dependent protein kinase catalytic subunit can decrease indels and increase precise genome editing outcomes through homology-directed repair (Robert et al., 2015; Canny et al., 2018; Riesenberg and Maricic, 2018; Riesenberg et al., 2019). While some work has been done to determine which DNA repair enzymes are responsible for the formation of small indels (Hussmann et al., 2021), less is known about the factors that promote large deletions. Recently, a study that used a clonal library of embryonic stem cells deficient for DNA repair genes found that inhibition of NHEJ-mediating enzymes increased frequencies of large deletions, while inhibition of microhomology-mediated end joining-mediating proteins decreased them (Kosicki et al., 2022). Of course, it is crucial to ensure that temporarily inhibiting DNA repair enzymes does not affect other regions of the cell’s genome. Another study has shown that the presence of an HDR template such as a single-stranded oligodeoxynucleotide or AAV donor can reduce the frequency of large deletions by 50%–80% (Wen et al., 2021), emphasizing the importance of testing unintended editing outcomes in the context of both the nuclease and the DNA donor.

While Cas9 nuclease technology continues to improve, recent editing tools replace this nuclease with a nickase to introduce single base pair changes or small site-specific modifications, most commonly in the form of single or dual nickase editing (Ran et al., 2013), base editing (Komor et al., 2016), or prime editing (Anzalone et al., 2019). Although these tools avoid formation of DSBs and likely reduce the frequency of large-scale genomic rearrangements, there is still the possibility of unintended off-target activity. In the case of base and prime editors, this arises from the tethering of Cas9 with deaminases and reverse transcriptases, respectively. In fact, some studies have reported that base editors can initiate off-target activity at sites with little homology to the gRNA (Jin et al., 2019; Zuo et al., 2019; Lei et al., 2021) and in a significant proportion of cellular mRNA (Grünewald et al., 2019a). In addition, base editor-induced modifications are often single nucleotide variants which are more difficult to detect by next-generation sequencing than localized indels introduced by traditional CRISPR nucleases. Ongoing efforts continue to engineer improved versions of these base editors to reduce off-target activity (Rees et al., 2017; Grünewald et al., 2019b; Li et al., 2022).

The above advances primarily concern the editing tools themselves, which is most likely to boost on-target effects and reduce unintended off-target consequences. However, these improvements will likely have limited impact on the ratio of on-tissue to off-tissue activity following *in vivo* delivery of editing tools. Toward this end, many groups are working to improve specificity of the delivery modalities themselves. This includes screening for vectors or nanoparticles that have specific tissue tropisms, such as those optimized to cross the blood-brain-barrier, to transduce vascular tissue, and more (White et al., 2004; Choudhury et al., 2016; Sago et al., 2018; Boehnke et al., 2022). There also are efforts to conjugate cell type-specific antibodies to delivery vectors to improve targeted *in vivo* delivery (Tombácz et al., 2021). While preliminary, this approach may be an effective means to improve on-tissue editing when injecting delivery vectors systemically.

In the early stages of development, but with great translational potential, are strategies to encode logic into cells (i.e., to introduce DNA code capable of responding to a given cellular state). As with CRISPR, many of these efforts use RNA-based homology to facilitate downstream expression of transgenes in the presence of a user-defined RNA sequence (Green et al., 2014, 2017; Kaseniit et al., 2022). Several proof-of-concept studies demonstrated that this technology could be used to encode complex logic into cells, such as multi-input OR, AND, and NOT gates. While much of this work was done in *E. coli* or human cell lines, if an analogous system was ported to clinically relevant primary cells it could allow genome editing tools to only be expressed in cells with a particular gene expression profile—effectively reducing or eliminating off-tissue activity.

## Concluding remarks

All the above efforts have been aimed at reducing unintended off-target and off-tissue activity. However, because millions or billions of cells are transplanted with *ex vivo* therapies, and billions or trillions of cells may be transduced with *in vivo* delivery vectors, any degree of unintended activity has the potential to be deleterious. Jennifer Doudna stated that one day she hopes to see CRISPR become a “standard of care” (Jennifer Doudna and William Kearney). If this is ever to become a reality, how do we make these therapies safe enough to be delivered routinely to patients?

While any manipulation to the genome opens the possibility for unwanted genetic events, we believe advances in off-target/off-tissue detection methods and improvements in genome editing tools and delivery modalities will ultimately allow personalized medicine to become a reality. As the development of advanced tools allows us to introduce increasingly complex features (and even logic) into cells, we will likely have to establish increasingly complex safety mechanisms as well. These may include automatic or inducible safety switches that provide a necessary safeguard in the instance of adverse clinical events (Di Stasi et al., 2011; Liang et al., 2018; Martin et al., 2020). And while initial data from CRISPR-based therapies in the clinic (both *ex vivo* and *in vivo*) has shown incredible promise, as greater numbers of patients receive genome editing treatments, we must ensure that editing *safety* keeps pace with *efficacy*. If CRISPR is ever to become the standard-of-care, then all of us—basic biologists, synthetic biologists, bioinformaticists, and clinicians—will have to combine efforts to ensure that genome editing therapies are as safe as possible.
