# Corrigendum: CRISPR nuclease off-target activity and mitigation strategies

**DOI:** 10.3389/fgeed.2022.1112956

**Published:** 2023-01-20

**Authors:** Beeke Wienert, M. Kyle Cromer

**Affiliations:** ^1^ Graphite Bio, Inc., South San Francisco, CA, United States; ^2^ Department of Surgery, University of California, San Francisco, San Francisco, CA, United States; ^3^ Department of Bioengineering and Therapeutic Sciences, University of California, San Francisco, San Francisco, CA, United States; ^4^ Eli and Edythe Broad Center for Regeneration Medicine, University of California, San Francisco, San Francisco, CA, United States

**Keywords:** gene therapy, off-target activity, *in vivo* delivery, genome editing, CRISPR/Cas9, next-generating sequencing

In the published article, “Dobbs et al., 2022” was cited incorrectly in the article. The citation has now been inserted at the correct location in the section, “**Methods to find off-targets sites in genome editing applications**”, paragraph three, and should read: “These methods vary widely in their approach and even starting material, using cell-free genomic DNA *in vitro* **(** **Kim et al., 2015** **;** **Cameron et al., 2017** **;** **Tsai et al., 2017** **;** **Kim and Kim, 2018** **;** **Lazzarotto et al., 2020** ), in intact live cells *ex vivo* (Crosetto et al., 2013; Tsai et al., 2015; Yan et al., 2017; Wienert et al., 2019; 2020; Zhu et al., 2019; Dobbs et al., 2022), and *in vivo* animal models (Akcakaya et al., 2018; Wienert et al., 2019; Liang et al., 2022).”

The authors apologize for this error and state that this does not change the scientific conclusions of the article in any way. The original article has been updated.

